# Extreme Precipitation and Flooding Contribute to Sudden Vegetation Dieback in a Coastal Salt Marsh

**DOI:** 10.3390/plants10091841

**Published:** 2021-09-05

**Authors:** Camille LaFosse Stagg, Michael J. Osland, Jena A. Moon, Laura C. Feher, Claudia Laurenzano, Tiffany C. Lane, William R. Jones, Stephen B. Hartley

**Affiliations:** 1 U.S. Geological Survey, Wetland and Aquatic Research Center, Lafayette, LA 70506, USA; mosland@usgs.gov (M.J.O.); lfeher@usgs.gov (L.C.F.); jonesb@usgs.gov (W.R.J.); hartleys@usgs.gov (S.B.H.); 2 U.S. Fish and Wildlife Service, Unified Regions 6&7, Upper Gulf Coast Zone, Winnie, TX 77665, USA; jena_moon@fws.gov; 3 Cherokee Nation System Solutions under Contract to U.S. Geological Survey, Wetland and Aquatic Research Center, Lafayette, LA 70506, USA; claurenzano@contractor.usgs.gov; 4 U.S. Fish and Wildlife Service, Ecological Services, Jacksonville, FL 32256, USA; tiffany_lane@fws.gov

**Keywords:** extreme climatic events, extreme precipitation, Hurricane Harvey, tropical cyclones, coastal wetlands, ecosystem collapse, regime shift, ecological thresholds, sudden vegetation dieback

## Abstract

Climate extremes are becoming more frequent with global climate change and have the potential to cause major ecological regime shifts. Along the northern Gulf of Mexico, a coastal wetland in Texas suffered sudden vegetation dieback following an extreme precipitation and flooding event associated with Hurricane Harvey in 2017. Historical salt marsh dieback events have been linked to climate extremes, such as extreme drought. However, to our knowledge, this is the first example of extreme precipitation and flooding leading to mass mortality of the salt marsh foundation species, *Spartina alterniflora*. Here, we investigated the relationships between baseline climate conditions, extreme climate conditions, and large-scale plant mortality to provide an indicator of ecosystem vulnerability to extreme precipitation events. We identified plant zonal boundaries along an elevation gradient with plant species tolerant of hypersaline conditions, including succulents and graminoids, at higher elevations, and flood-tolerant species, including *S. alterniflora*, at lower elevations. We quantified a flooding threshold for wetland collapse under baseline conditions characterized by incremental increases in flooding (i.e., sea level rise). We proposed that the sudden widespread dieback of *S. alterniflora* following Hurricane Harvey was the result of extreme precipitation and flooding that exceeded this threshold for *S. alterniflora* survival. Indeed, *S. alterniflora* dieback occurred at elevations above the wetland collapse threshold, illustrating a heightened vulnerability to flooding that could not be predicted from baseline climate conditions. Moreover, the spatial pattern of vegetation dieback indicated that underlying stressors may have also increased susceptibility to dieback in some *S. alterniflora* marshes.Collectively, our results highlight a new mechanism of sudden vegetation dieback in *S. alterniflora* marshes that is triggered by extreme precipitation and flooding. Furthermore, this work emphasizes the importance of considering interactions between multiple abiotic and biotic stressors that can lead to shifts in tolerance thresholds and incorporating climate extremes into climate vulnerability assessments to accurately characterize future climate threats.

## 1. Introduction 

As Earth’s climate continues to change, life on our planet will be determined not only by higher temperatures [1] and rising sea levels [2], but also by changes in the frequency and intensity of climatic events [3]. For example, precipitation events are expected to intensify as heavy rainfall is condensed into shorter periods of time [4]. Hurricanes are expected to become more extreme [5,6], moving more slowly, with faster winds [7] and heavier precipitation [8]. These climate extremes threaten ecological communities [9,10] by lowering the resilience of foundation species [11], which, in the most extreme cases, can lead to ecological regime shifts or ecosystem collapse [12]. Extreme ecological responses, such as large-scale die-off of foundation species, have been linked to climate extremes around the world. For example, in Australia, a series of extreme climatic events were characterized by the widespread demise of critical marine habitat-forming organisms including corals, kelps, seagrasses, and mangroves [13]. Extreme climatic events, triggered by extreme hurricanes and drought, have led to the massive die-off of foundation species in coastal wetlands, including mangrove trees [14] and salt marsh grasses [15]. 

Given future projections of more frequent and intense climatic extremes, it is critical to understand the mechanisms governing catastrophic ecosystem responses. This information will allow researchers to predict ecosystem shifts and ecosystem collapse, and can help natural resource managers develop conservation plans and strategies that may mitigate future threats of climate change to these valuable ecosystems. Although many studies have characterized the effects of changing climatic means, ecological responses to climate extremes may not follow trends quantified under baseline climate conditions [9]. Therefore, excluding extreme events from ecological assessments may critically underestimate vulnerability to future climate conditions [16].

To provide an indicator of ecosystem vulnerability to climate extremes, we investigated the relationship between extreme abiotic conditions and large-scale plant mortality of a coastal wetland foundation species. In 2017, Hurricane Harvey, a catastrophic Category 4 tropical cyclone, made landfall along the northern Gulf of Mexico, stalling for several days and flooding the coastal regions of Texas (USA) with storm surge [17] and extreme levels of precipitation [18]. Following the extreme flooding event that persisted for up to two months (Sargent, TX Station ID: 8772985) [19], sudden dieback of the salt marsh foundation species, *Spartina alterniflora* (hereafter, *Spartina* or *S. alterniflora*), was observed in some coastal marshes of Texas that were impacted by extreme precipitation and freshwater flooding (Figure S1). 

Hydrology is among the most important factors controlling coastal wetland function and sustainability. Flooding is known to define plant zonation based upon the biological tolerances of individual species to flood depth, duration, and frequency [20,21,22]. Thus, changes in flooding can cause shifts in wetland community composition reflecting a dynamic ecological response that can also be cyclic in nature. For example, in the mid-coast region of Texas along the northern Gulf of Mexico, cycles of drought and ample precipitation are associated with shifts in salinity regimes, inundation regimes, and coastal wetland foundation plant species. During dry periods with low freshwater inflows, coastal wetlands are dominated by drought- and salt-tolerant succulent plant species, whereas during wet periods, coastal wetlands are dominated by more flood-tolerant graminoid plant species [23,24,25]. This dynamic response illustrates ecosystem sensitivity and resilience to environmental perturbations; however, extreme changes in environmental conditions may surpass resilience thresholds leading to ecosystem collapse [26]. 

To better understand the mechanisms of baseline and extreme flooding on large-scale *S. alterniflora* marsh dieback, we tested the following hypotheses: (1) flooding controls marsh plant zonation along an elevation gradient, where species tolerant of hypersaline conditions occur at higher elevations and flood-tolerant species occur at lower elevations; (2) the relationship between plant cover and elevation is nonlinear, and the conversion of vegetated marsh to open water occurs beyond an abrupt elevation threshold; and (3) the sudden widespread dieback of *S. alterniflora* following extreme precipitation and flooding occurred above the elevation threshold for wetland collapse.

## 2. Methods

### 2.1. Study Location and Experimental Design

**Figure 1:**
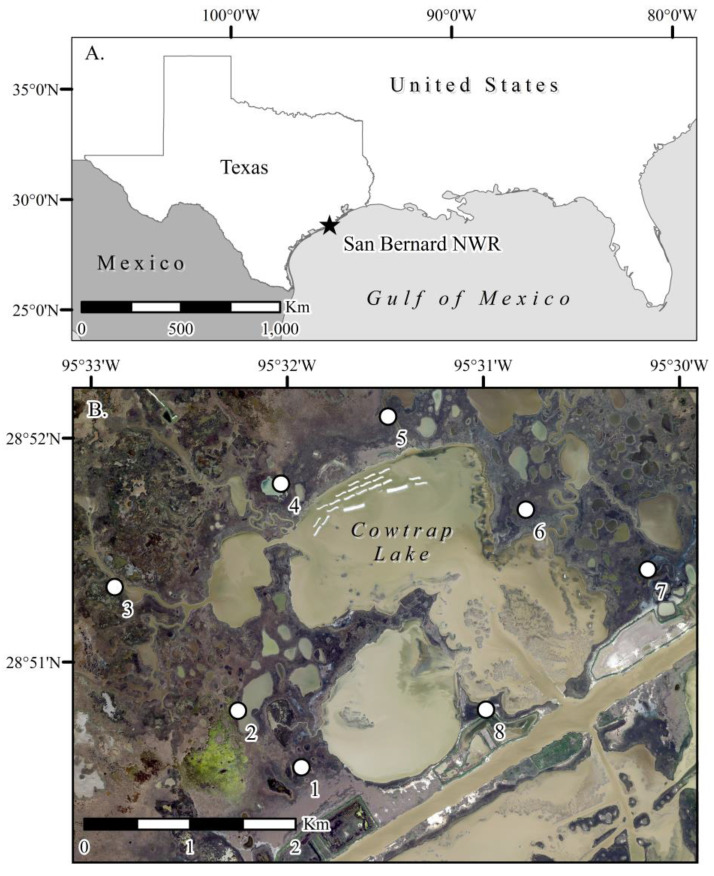
(**A**). Location of San Bernard National Wildlife Refuge (NWR) along the northern Gulf of Mexico coast (Texas, USA), and (**B**). Location of the eight salt marsh study sites (numbered circles) within San Bernard NWR, surrounding an area called Cowtrap Lake.

The study was conducted in the San Bernard National Wildlife Refuge (NWR), which is located along the northern Gulf of Mexico, in the mid-coast region of Texas (Figure 1). 

San Bernard NWR extends across 22,000 hectares, containing habitats ranging from bottomland forests along the Brazos River and San Bernard River seaward to tidal fresh marshes and salt marshes bordering the Gulf of Mexico. This study focused on the salt marsh habitat, where large areas of *S. alterniflora* dieback were observed in 2017, following Hurricane Harvey. In addition to *S. alterniflora* dieback, the salt marshes contained large areas of surviving healthy *S. alterniflora*, as well as other graminoid plants, including *Distichlis spicata*, and several succulent plant species, including *Batis maritima*, *Lycium carolinianum*, *Monanthochloe littoralis*, *Borrichia frutescens*, and *Salicornia depressa*.

Eight study sites with prominent occurrence of *S. alterniflora* dieback were selected within the salt marsh habitat. Within each site, ground-collected data were used to define cover categories characterizing dominant plant communities, *S. alterniflora* dieback zones, and areas of open water (Figure S2). Common to all sites, four cover categories were defined, which included “Open Water”, “*Spartina* Dieback”, “*Spartina*”, and “*Distichlis* + Succulents” (Figure S3). Open Water was defined as an area of open water of at least 4 m^2^. The *Spartina* Dieback cover category was devoid of live vegetation, containing standing dead *S. alterniflora*, or “stubble”, which is a term used in the salt marsh dieback literature. The *Spartina* category contained healthy live *S. alterniflora*, and the “*Distichlis* + Succulents” category was heterogeneous, characterized by distinct patches of *D. spicata* monocultures, succulent-only species, and a mixture of *D. spicata* and multiple succulent species. Except for the “*Distichlis* + Succulents” category, five plots were established for data collection within each cover category. Because the “*Distichlis* + Succulents” category was not homogenous, five plots were established in each of the three patch types, for a total of 15 plots per site within this cover category. In total, 225 plots were established among the eight sites. Plot location was determined by walking in the direction of a randomly-selected azimuth for at least 15 meters and until the first encounter of a patch within the target cover category. This ensured that all plots within each cover category were at least 15 m apart.

### 2.2. Remote Data Collection

To compare differences in surface vegetation condition within the *Spartina* Dieback areas before and after Hurricane Harvey, we used Google Earth Engine to calculate Normalized Difference Vegetation Index (NDVI) values derived from Landsat 8 and Landsat 7 atmospheric-corrected surface reflectance imagery collected between 2015 and 2019, resulting in the collection of 164 unique Landsat scenes [27]. Landsat surface reflectance imagery includes a pixel quality assessment band where each pixel is assigned an integer value that represents surface, atmospheric, and sensor conditions that can affect pixel quality [28]. In order to reduce the influence of pixels containing cloud cover, cloud shadows, or water, we used the pixel quality assessment band of each Landsat image to select only pixels identified as “clear terrain, low-confidence cloud, low-confidence cirrus” (pixel quality values 322 and 66 in Landsat 8 and Landsat 7, respectively) [29]. For each scene, NDVI was calculated as the normalized difference of the near-infrared and infrared bands, corresponding to bands 5 and 4 for Landsat 8 scenes, respectively, and bands 5 and 6 for Landsat 7 scenes, respectively. We used the coordinates of the 40 individual *Spartina* Dieback plots to extract the NDVI values from the overlying pixels in each scene. We then limited our comparisons of NDVI to values collected from imagery obtained between June and August of each year, because this time period represents the peak of the growing season for marshes in the Northern Gulf of Mexico. 

### 2.3. Field Data Collection

Within each 0.25 m^2^ plot (i.e., 0.5 m × 0.5 m), we measured vegetation canopy cover, canopy height, and surface elevation. Canopy cover for each species was visually estimated as a percent (0–100% cover), and visual estimation was performed by the same two researchers (C. Stagg and M. Osland) for consistency across all plots. We also measured mean canopy height for each species. Bare ground was calculated as the difference between 100% and the sum of % cover of all species within the plot (Table S1). Soil surface elevation was measured using Real-Time Kinematic (RTK) methods [30] with a Trimble R10 Global Navigation Satellite System (Trimble Navigation Limited, Sunnyvale, CA, USA) [31] in combination with a real-time Continuously Operating Reference Station (CORS) network developed by the Texas Department of Transportation. This methodology provides surface elevation data rectified to the North American Vertical Datum of 1988 (NAVD88). Upon initiation and completion of the survey, the Global Navigation Satellite System equipment was calibrated with an established elevation monument with a 95% confidence interval of 1.2 cm (Station DP0702) [32], and data was post-processed using Trimble Business Center 2.5 software (Trimble Navigation Limited, USA). Global Navigation Satellite System elevation data points were derived from 3 minute observations in each plot for a total of 225 3 minute elevation data points. 

### 2.4. Data Analyses

Prior to analyses of the Landsat-derived NDVI data, we converted the plot-level NDVI values from each date to site-level means, resulting in a data set with 251 site-level NDVI values calculated from both Landsat 8 and Landsat 7 imagery [33]. NDVI values from the 2015–2017 growing seasons were assigned to the pre-Harvey category, whereas NDVI values from the 2018–2019 growing seasons were assigned to the post-Harvey category. We used a paired *t*-test to compare the mean site-level NDVI values in the years before and after Hurricane Harvey. The pre- or post-Harvey category was the dependent variable and the site-level NDVI value was the independent variable. 

To compare field-collected surface elevations between cover categories, we used an analysis of variance (ANOVA) in combination with Tukey post hoc tests. Site-level canopy height and species cover means and standard errors were calculated for each cover category.

We conducted a sigmoidal regression analysis to examine the relationship between surface elevation and the combined cover of the “*D. spicata* + Succulent” species at each site. The elevation boundaries of the “*D. spicata* + Succulent” zone (i.e., the zonal boundaries) were calculated using the local maximum peak of the second derivative of the sigmoidal equation (lower elevation boundary) and the highest elevation for this zone (higher elevation boundary). 

We conducted separate nonlinear regressions to examine the relationships between surface elevation and the cover of *Spartina* or *Spartina* Dieback using normal probability distribution equations. The elevation boundaries of the *Spartina* or *Spartina* Dieback zones (i.e., the zonal boundaries) were then calculated as the area between the local maximum peaks of the second derivative of the normal probability distribution equations. 

To examine the relationship between surface elevation and plant cover, we conducted a sigmoidal regression analysis using data from all sites. To identify the baseline elevation threshold that separated open water from marsh vegetation (excluding *Spartina* Dieback), we calculated the local maxima of the first derivative (T) of the sigmoidal equation, which represents the point of the maximum rate of change. A threshold transition zone was determined from the area of maximum rate of change (AMRC), which was calculated as the area between the local maximum and minimum peaks of the second derivative of the sigmoidal equation [34,35]. All data analyses were performed in R [36].

## 3. Results

The mean site-level NDVI of *Spartina* Dieback plots was significantly greater in the pre-Harvey time period (mean = 0.50, se = 0.01) compared to the post-Harvey time period (mean = 0.37, se = 0.02) (t(7) = 7.66, *p* < 0.001, Figure S1B). 

**Figure 2:**
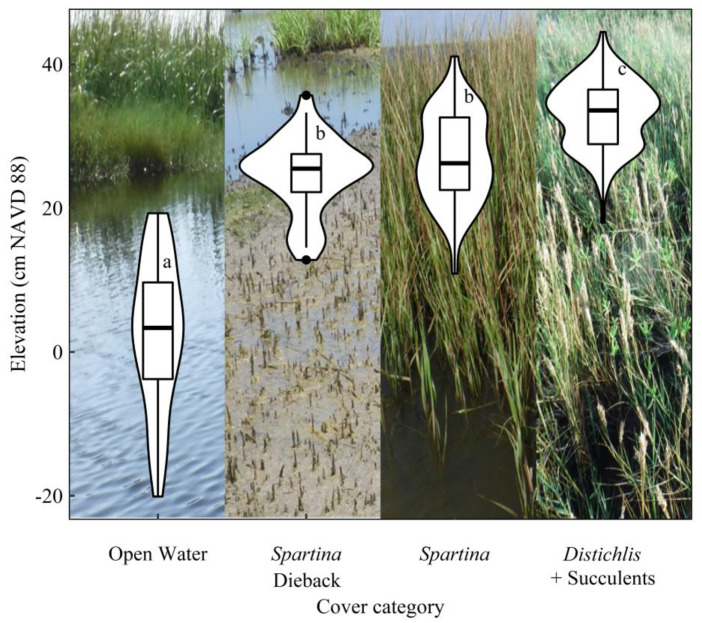
Box plots of the elevation for each cover category. Lower and upper box boundaries represent the 25th and 75th percentile, respectively, whereas the lines inside the boxes represent the median. The lower and upper error bars represent the 10th and 90th percentile, respectively. The outer ‘violin’ shapes show the probability density of the elevation values in each cover category. Groups with the same letter are not significantly different (*p* > 0.05). Elevation data are rectified to the North American Vertical Datum of 1988 (NAVD88).

**Figure 3:**
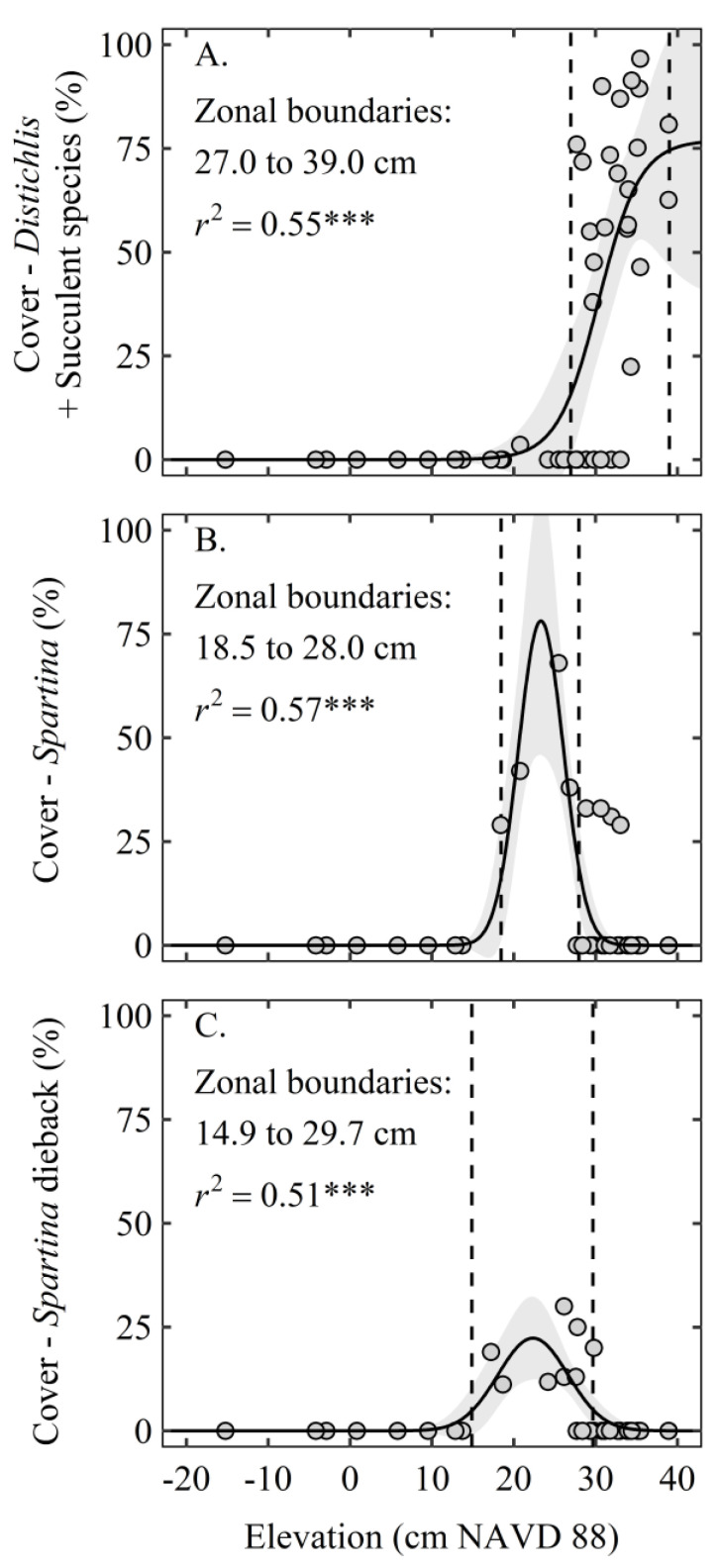
The relationship between elevation and the cover of (**A**). *Distichlis* + Succulents (*Distichlis spicata, Batis maritima, Lycium carolinianum, Monanthochloe littoralis, Borrichia frutescens, and Salicornia depressa*), (**B**). *Spartina*, and (**C**). *Spartina* Dieback. The dashed lines represent the elevation zonal boundaries and the gray shaded areas represent the 95% confidence intervals of the regressions. *** indicates *p* < 0.001. Elevation data are rectified to the North American Vertical Datum of 1988 (NAVD88).

Generally, species in the *Distichlis* + Succulents cover type occurred at a significantly higher elevation compared to the *Spartina* and *Spartina* Dieback cover types, and Open Water occurred at the lowest elevation (Figure 2). Nonlinear regressions of plant cover and surface elevation explained significant variation for each cover type and revealed that plant communities occupied specific zones along the elevation gradient that were characterized by peak plant performance within a certain elevation range (i.e., zonal boundaries) (Figure 3A–C). Peak canopy cover of *D. spicata* and succulent species occurred within the zonal boundaries of 27.0 and 39.0 cm NAVD88. The healthy *Spartina* zone occurred at a lower elevation range, between 18.5 and 28.0 cm NAVD88, which overlapped with *Spartina* Dieback zonal boundaries, 14.9 to 29.7 cm NAVD88.

**Figure 4:**
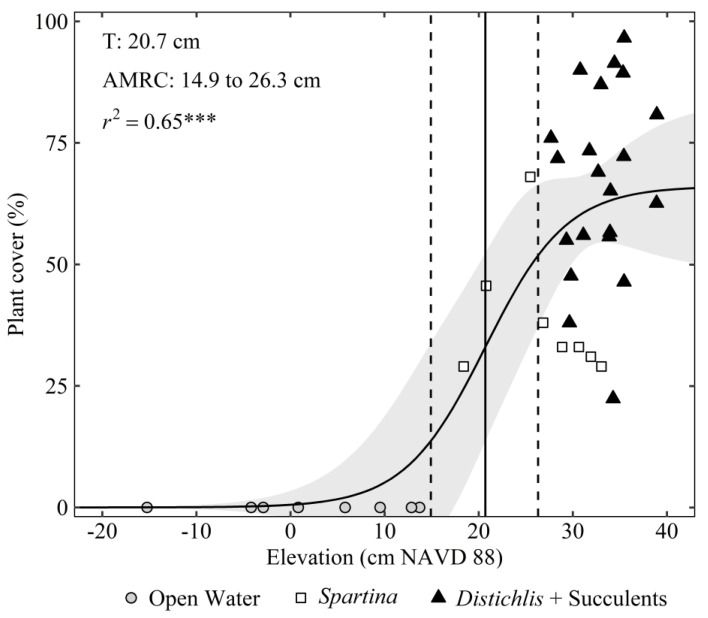
The sigmoidal relationship between elevation and salt marsh plant cover. The discrete elevation threshold (i.e., threshold [T]) is represented by a solid line and the dashed lines represent the elevation threshold zone boundaries (i.e., area of the maximum rate of change [AMRC]). The light grey shaded area represents the 95% confidence intervals of the regression. The symbols reflect three of the four vegetation categories. The *Spartina* Dieback category is not included. *** = *p* < 0.001. Elevation data are rectified to the North American Vertical Datum of 1988 (NAVD88).

**Figure 5:**
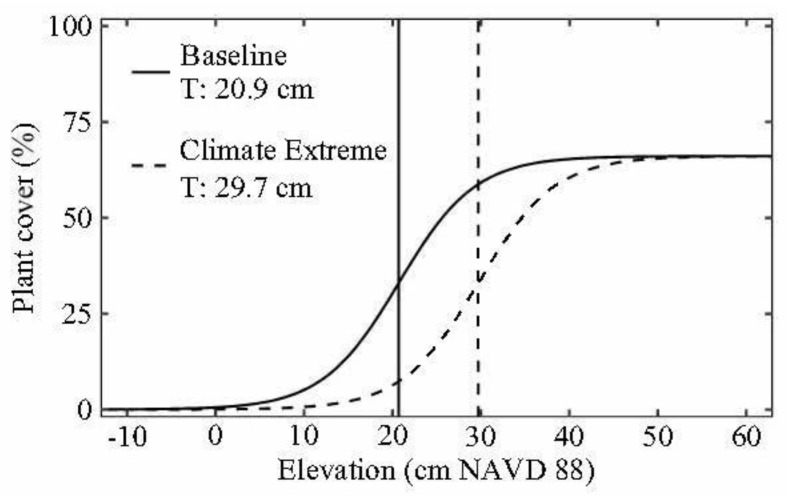
The shift in the elevation threshold (T) for the marsh-to-open water transition following Hurricane Harvey. The baseline threshold is represented by the solid line (Baseline T: 20.9 cm), and the post-Harvey threshold is represented by the dashed line (Climate Extreme T: 29.7 cm). Elevation data are rectified to the North American Vertical Datum of 1988 (NAVD88).

Across all sites, the relationship between plant cover and surface elevation was sigmoidal in nature, with an elevation threshold of 20.7 cm NAVD88 separating the transition from vegetated marsh to open water (Figure 4). As elevation declined, the transition from the *Distichlis* + Succulents zone to the *Spartina* zone was characterized by a gradual decline in plant cover until the upper boundary of the threshold zone, or area of maximum rate of change (AMRC), was reached at 26.3 cm NAVD88. *Spartina* occupied the threshold zone between 26.3 and 14.9 cm NAVD88, where small decreases in elevation led to large declines in *Spartina* cover. Beyond the lower threshold boundary of 14.9 cm NAVD88, there was a steep decline in *Spartina* cover representing an abrupt transition from vegetated marsh to open water. *Spartina* Dieback zonal boundaries were observed at elevations of up to 9 cm above the pre-Harvey elevation threshold for the marsh to open water transition (Figure 5). Results of the nonlinear regression analyses are shown in the Supplementary Material, Tables S2 and S3.

## 4. Discussion

Following the recent record-breaking 2020 Atlantic Ocean hurricane season, noted for the highest number of landfalling storms in U.S. history [37], the growing threat of hurricanes to coastal ecosystems is clear. As sea surface temperatures continue to increase, both the frequency and intensity of major hurricanes are expected to rise [3]. A key aspect of increasingly severe storms is more extreme precipitation. Intense rainfall events associated with hurricanes can cause significant flooding, as was observed with Hurricane Harvey in 2017 [38]. Along the northern Gulf of Mexico coast and the U.S. southeastern Atlantic coast, hurricane-related precipitation events contribute a significant fraction to annual maximum precipitation [39]. Indeed, hurricane-related rainfall totals along the Gulf of Mexico are among the highest in the world (100–150 mm y^-1^) [40]. Furthermore, some studies suggest that hurricane-related precipitation intensity (amount per unit time) is also increasing [41], which may lead to unprecedented impacts to coastal ecosystems. 

Many coastal wetlands have adapted to regular hurricane disturbances over time, which can have both damaging and beneficial effects [42], shaping ecosystem structure and function through impacts to wetland geomorphology, vegetation structure, hydrology, and nutrient cycling [43,44,45,46,47]. However, increasingly intense storms may generate extreme abiotic conditions that could make coastal wetland recovery difficult or impossible [48,49,50]. For example, extreme precipitation events can cause excessive and prolonged flooding, which has been known to trigger regime shifts in coastal wetlands causing a transition from vegetated wetland to open water [51]. 

Following the extreme precipitation and flooding event associated with Hurricane Harvey, observations of *S. alterniflora* mortality were consistent with patterns of sudden vegetation dieback, a massive die-off event that occurs in coastal salt marshes and is characterized by the acute mortality of *S. alterniflora* (within a single growing season) with conversion of vegetated marsh to mudflat or open water [52]. Although most observations of sudden vegetation dieback in salt marshes have been associated with severe drought [53], large-scale *S. alterniflora* mortality following Hurricane Harvey indicates a link between extreme flooding and sudden vegetation dieback in *S. alterniflora* marshes. 

*Spartina alterniflora* tolerates deep and prolonged flooding [54], and subsequently often inhabits lower elevation zones compared to other salt marsh vegetation [21,22]. However, even with *S. alterniflora*, excessive flooding beyond a tolerance threshold can inhibit growth and cause complete plant mortality [55]. Ecological thresholds are common in coastal wetlands and represent a nonlinear response to abiotic conditions [56]. In the current study, we quantified the elevation threshold where relatively small declines in elevation resulted in significant deterioration in *S. alterniflora* plant performance, and ultimately the conversion from vegetated marsh to open water. This elevation threshold represents the historical, baseline maximum tolerance for flooding stress beyond which *S. alterniflora* cannot survive. However, the elevation zonal boundaries of *S. alterniflora* dieback zones were up to 9 cm higher than the baseline elevation threshold that defines the conversion from healthy vegetated marsh to open water, illustrating a shift in the flood tolerance threshold following the extreme precipitation event (Figure 5). These findings not only illustrate an important difference in the ecological response to baseline “press” conditions compared to extreme “pulse” conditions [57,58], but also highlight the enhanced vulnerability of these ecosystems to future climate conditions. Understanding the mechanisms that define these ecological thresholds is critical to predicting the vulnerability of these ecosystems to future climate threats, including extreme precipitation and flooding. 

Hidden players (sensu Thompson et al., 2001) [59], such as microbial or fungal pathogens, can contribute to underlying stress that can erode the resilience of an ecosystem, effectively lowering the tolerance threshold and rendering it vulnerable to an acute disturbance, like extreme drought or flooding [60,61]. In the current study, it is likely that the areas of *S. alterniflora* dieback were weakened by an underlying stressor, making them more sensitive to extreme precipitation and flooding associated with Hurricane Harvey. In coastal salt marshes, several biotic stressors have been linked to sudden vegetation dieback, including marine crustaceans [62], marine gastropods [63], and fungal pathogens [64]. Because biotic stressors can interact additively or synergistically with abiotic stressors [65], future research may benefit from investigating the prevalence and impact of consumers and pathogens in *S. alterniflora* marshes impacted by extreme flooding. 

Although the complexity of mechanisms controlling extreme climatic events makes threshold identification more difficult [16], the implications of underestimating the vulnerability of these valuable ecosystems to future climate threats emphasize the importance of further research on this topic. Collectively, our results highlight a new mechanism of sudden vegetation dieback in *S. alterniflora* marshes that is triggered by extreme precipitation and flooding. Furthermore, this work emphasizes the importance of considering interactions between multiple abiotic and biotic stressors that can lead to shifts in tolerance thresholds and incorporating climate extremes into climate vulnerability assessments to accurately characterize future climate threats.

## Data Availability

All data is publicly available as a USGS Data Release on ScienceBase.gov access on 1 May 2021. Stagg, C.L., Osland, M.J., Moon, J.A., Feher, L.C., Laurenzano, C., Lane, T.C., Jones, W.R., and Hartley, S.B., 2021, Coastal wetland vegetation and elevation data characterizing a Sudden vegetation dieback event in San Bernard National Wildlife Refuge in 2019: U.S. Geological Survey data release, https://doi.org/10.5066/P92UFF8MK access on 1 May 2021.

## Supplementary Materials

The following are available online at https://www.mdpi.com/article/10.3390/plants10091841/s1, Figure S1: Cumulative daily precipitation and NDVI values associated with Hurricane Harvey. Figure S2: Study site drone imagery and land cover classification. Figure S3: Cover category photos. Table S1: Vegetation canopy height and species cover. Table S2: Sigmoidal regression results. Table S3: Normal probability distribution model results.
