# Supplementary material for: Extreme Precipitation and Flooding Contribute to Sudden Vegetation Dieback in a Coastal Salt Marsh
Source: Plants (Basel). 2021 Sep 5;10(9):1841. doi: 10.3390/plants10091841 (PMC8471974; doi:10.3390/plants10091841)
Supplement: Supplementary file 1 [file plants-10-01841-s001.zip › plants-1348115-supplementary.pdf]

Supplementary Materials

Extreme Precipitation and Flooding Contribute to Sudden Vegetation Dieback in a Coastal Salt Marsh

Stagg, Camille L.[1*], Osland, Michael J.[1], Moon, Jena A.[2], Feher, Laura C.[1], Laurenzano, Claudia[3], Lane, Tiffany C.[4], Jones, William R.[1], and Hartley, Stephen B.[1]

[1] U.S. Geological Survey, Wetland and Aquatic Research Center, Lafayette, LA, USA

[2] U.S. Fish and Wildlife Service, Unified Regions 6&7, Upper Gulf Coast Zone, Winnie, TX, USA

[3] Cherokee Nation System Solutions under contract to U.S. Geological Survey, Wetland and Aquatic Research Center, Lafayette, LA, USA

[4] U.S. Fish and Wildlife Service, Ecological Services, Jacksonville, FL, USA

*Corresponding Author: staggc@usgs.gov, Telephone 337-316-7544, 700 Cajundome Boulevard, Lafayette, LA 70506, U.S.A.

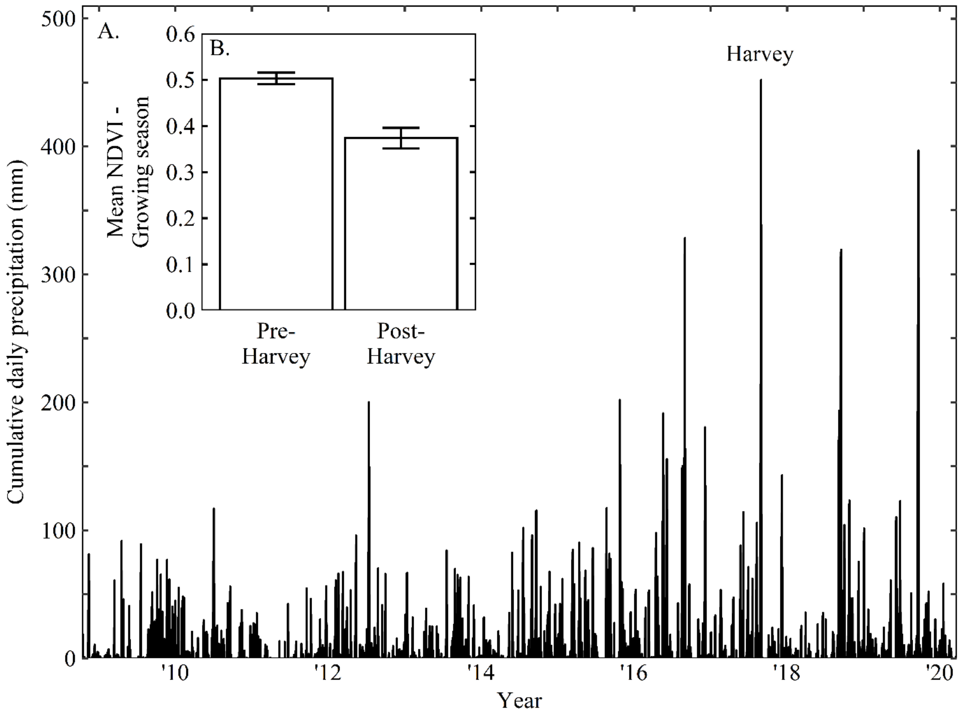

Figure S1. A. Cumulative daily precipitation near Freeport, Texas (USA; from Menne et al., 2012-NOAA Global Historical Climatology Network - Daily Station USC00413340), between 2009 to 2019. The high precipitation event associated with Hurricane Harvey between 17 August – 2 September, 2017 is labeled "Harvey". B. Mean site-level NDVI of the *Spartina alterniflora* dieback plots at San Bernard NWR during the growing season (June through August) of the years before Hurricane Harvey (pre-Harvey: 2015-2017) and after Hurricane Harvey (post-Harvey: 2018-2019). Site-level NDVI of the *S.*

alterniflora dieback plots was significantly greater in the pre-Harvey time period than the post-Harvey time period.

A.

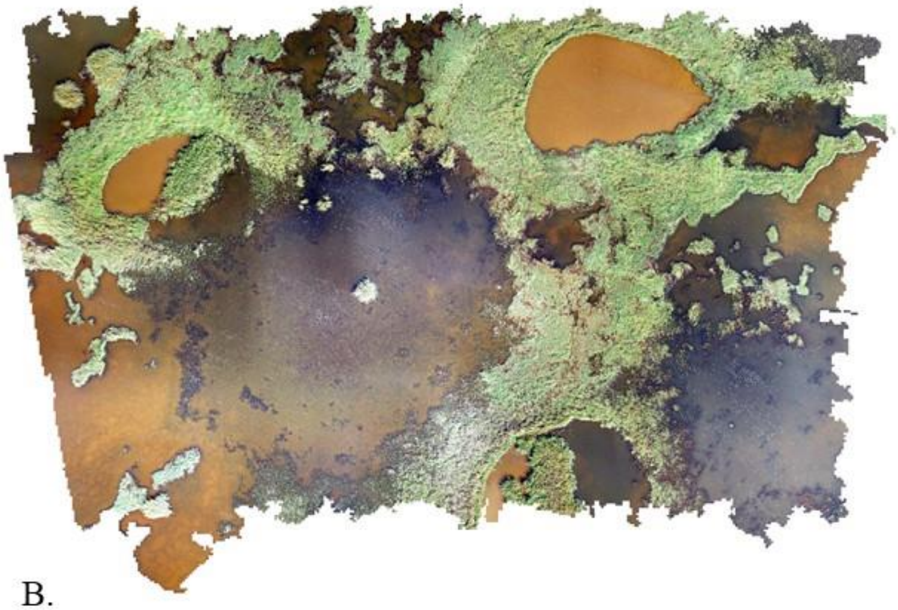

B.

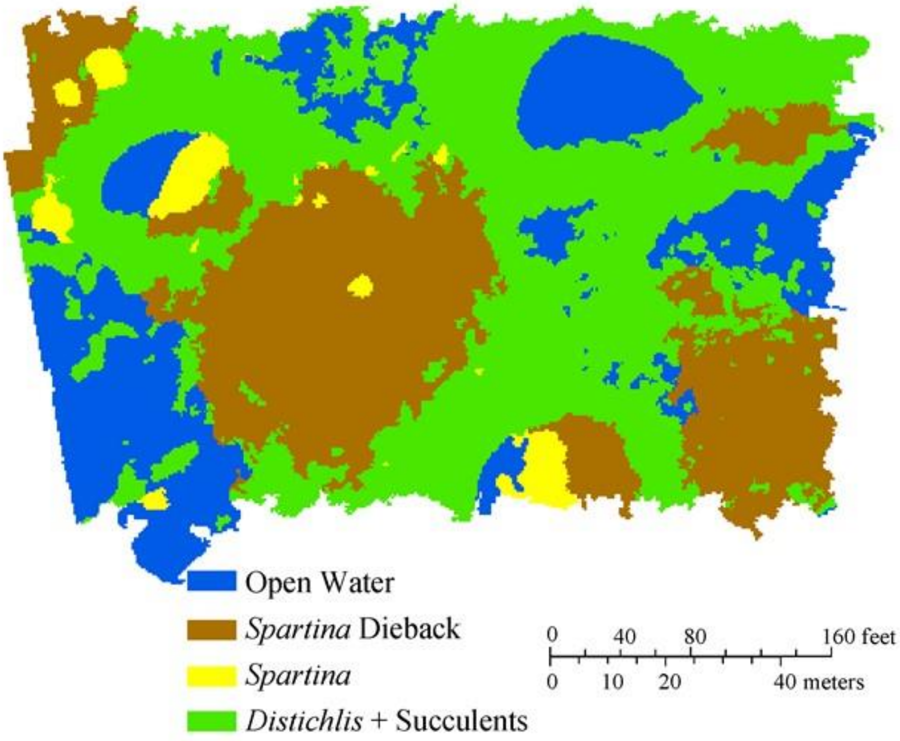

Figure S2. Example of salt marsh study site with A. drone imagery, and B. imagery classification identifying the four cover categories.

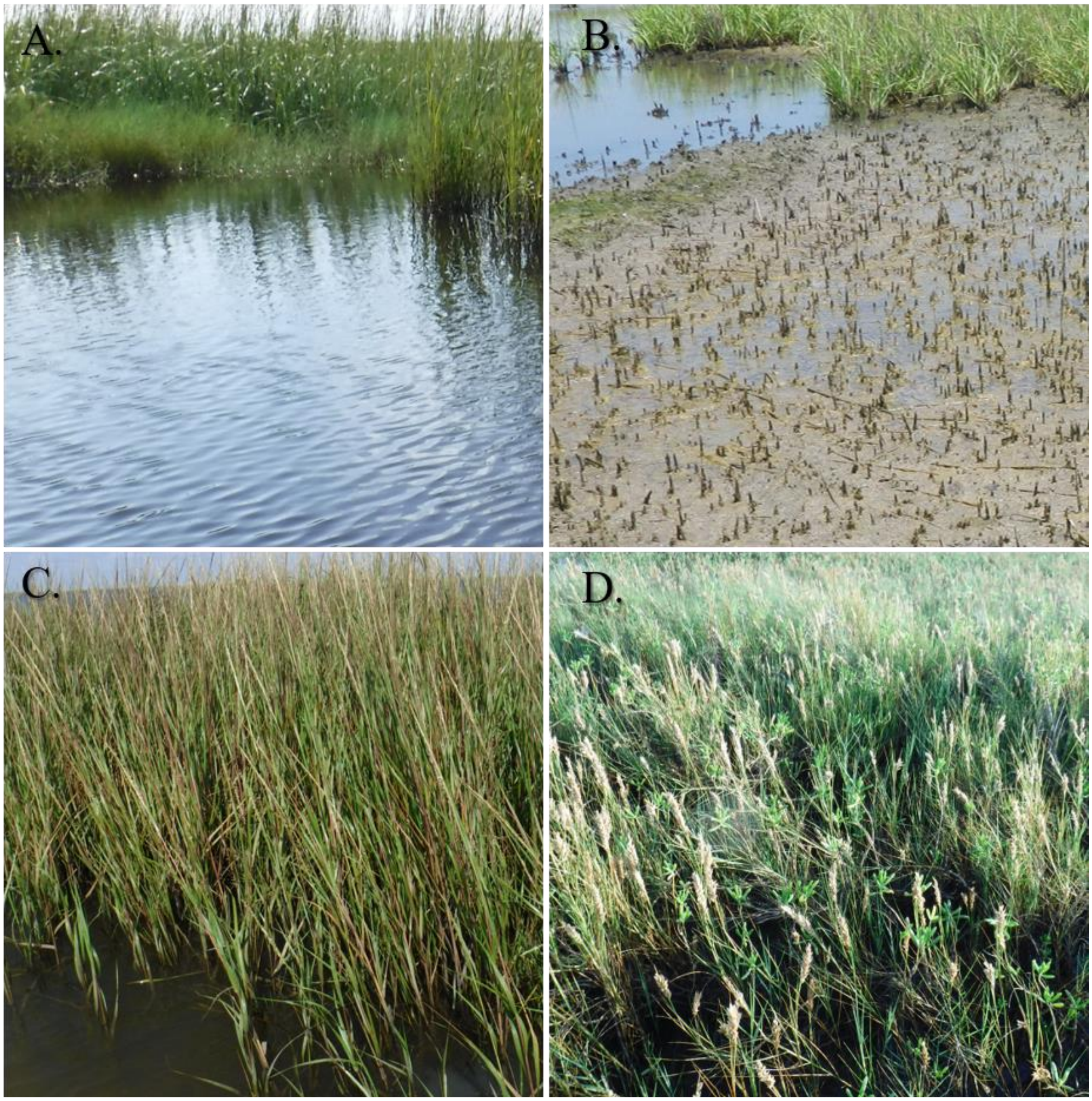

Figure S3. Photos of the four cover categories identified in each salt marsh site. A. Open Water, B. *Spartina* Dieback (note the dead stems called "stubble"), C. *Spartina*, and D. *Distichlis*+Succulents. Photo credit: Camille L. Stagg, public domain.

Table S1. Canopy height (cm) and species cover (%) for each of the four cover categories (rows). Cover category means (± SE) were estimated at the site level and included replicate plots.

Cover category	Canopy height (cm)		Species cover (%)															
			Disp		Bama		Lyca		Moli		Bofr		Sade		Spal	
	mean	se	mean	se	mean	se	mean	se	mean	se	mean	se	mean	se	mean	se
Open Water	0	0	0	0	0	0	0	0	0	0	0	0	0	0	0	0
Spartina Dieback	0.3	0.3	0	0	0	0	0	0	0	0	0	0	0	0	0	0
Spartina	137.2	5.3	0	0	0.4	0.4	0	0	0	0	0	0	0	0	37.9	4.6
Distichlis + Succulents	44.8	2.4	48.7	5.6	10.1	2	1	0.5	6	3.3	1	0.4	0.1	0.1	0	0

Species cover abbreviations as follows: Disp = *Distichlis spicata;* Bama = *Batis maritima;* Lyca = *Lycium carolinianum;* Moli = *Monanthochloe littoralis;* Bofr = *Borrichia frutescens;* Sade = *Salicornia depressa;* Spal = *Spartina alterniflora.*

Table S2. Results of non-linear (sigmoidal) regression models used to test relationships between % cover of the target cover categories and elevation. For the sigmoidal regression analyses, we used the equation $y(x) = \frac{a}{1+e^{-\frac{(x-c)}{b}}}$, where y = % cover of the target cover category, x = elevation, a = the asymptote, b = the function growth rate, and c = the function midpoint.

% Cover of the cover category (y)	p	a	b	c	$F_{(n,k)}$	r^2
Total plant cover	***	66.1	0.2	0.02	32.0 $_{(2, 36)}$	0.65
Distichlis + Succulents cover	***	76.6	0.3	0.03	25.8 $_{(2, 42)}$	0.55

Table S3. Results of the non-linear (normal probability distribution) models used to test relationships between the % cover of the target cover categories and elevation.

For the normal probability distribution regression analyses, we used the equation $y(x) = N \times \left(\frac{1}{\sqrt{(2\pi)}\sigma} e^{-(x-\mu)^2/2\sigma^2}\right)$, where y = % cover of the target cover category, x = elevation, μ = the mean of the normal probability distribution, σ = the standard deviation of the normal probability distribution, and N = the normalization factor.

% Cover of the cover category (y)	p	μ	σ	N	$F_{(n,k)}$	r^2
Spartina cover	***	0.2	0.03	5.4	22.2 $_{(2,36)}$	0.57
Spartina Dieback cover	***	0.2	0.04	2.4	17.8 $_{(2,36)}$	0.51
